# Extreme philopatry and genetic diversification at unprecedented scales in a seabird

**DOI:** 10.1038/s41598-021-86406-9

**Published:** 2021-03-25

**Authors:** D. K. Danckwerts, L. Humeau, P. Pinet, C. D. McQuaid, M. Le Corre

**Affiliations:** 1 Coastal Research Group, Department of Zoology and Entomology, Rhodes University, Grahamstown, 6140 South Africa; 2 UMR ENTROPIE (Université de La Réunion, IRD, CNRS, IFREMER, Université de Nouvelle-Calédonie), 15 Avenue René Cassin, CS 92003, 97744 Saint Denis Cedex 9, Ile de La Réunion France; 3 UMR PVBMT (Université de La Réunion, CIRAD), 15 Avenue René Cassin, CS 92003, 97744 Saint Denis Cecodex 9, Ile de La Réunion France; 4 Parc National de La Réunion, LIFE+ Petrels, 258 rue de la République, 97431 Plaine des Palmistes, Ile de La Réunion France; 5 Terres Australes Et Antarctique Françaises (TAAF), rue Gabriel Dejean, 97410 Saint-Pierre, Ile de La Réunion France

**Keywords:** Genetic variation, Population dynamics, Conservation biology

## Abstract

Effective conservation requires maintenance of the processes underlying species divergence, as well as understanding species’ responses to episodic disturbances and long-term change. We explored genetic population structure at a previously unrecognized spatial scale in seabirds, focusing on fine-scale isolation between colonies, and identified two distinct genetic clusters of Barau’s Petrels (*Pterodroma baraui*) on Réunion Island (Indian Ocean) corresponding to the sampled breeding colonies separated by 5 km. This unexpected result was supported by long-term banding and was clearly linked to the species’ extreme philopatric tendencies, emphasizing the importance of philopatry as an intrinsic barrier to gene flow. This implies that loss of a single colony could result in the loss of genetic variation, impairing the species’ ability to adapt to threats in the long term. We anticipate that these findings will have a pivotal influence on seabird research and population management, focusing attention below the species level of taxonomic organization.

## Introduction

A topic of critical importance in conservation biology concerns the mechanisms through which evolutionary changes occur, including the influence of population connectivity, and the implications of variation in connectivity on a species’ long-term survival and conservation needs^1,2^. Seabirds present several challenges to the generally accepted mechanisms of population differentiation in other vagile groups^3,4^ and, despite recent advances in genetic theory and approaches, the scales at which genetic differentiation exists among their populations remains extremely difficult to predict^5^. Seabirds have an almost unsurpassed flight potential and have been known to cross physical features that limit dispersal in less motile groups^6–8^. Consequently, one could expect seabirds to disperse freely among their breeding sites yet growing evidence suggests that the potential for long-distance movement is not always the best indicator of gene flow^9,10^. This implies that philopatry and other intrinsic barriers to dispersal (e.g. differences in breeding phenology) may have important roles in the evolution of seabird diversity and endemism^1,3^, and that predicting genetic differentiation among populations for management or systematic purposes requires the assessment of these factors^4^. Despite this, little is known about the scales at which intrinsic barriers to genetic dispersal operate with few studies focusing on fine-scale inter-colony observations.

Understanding population genetic differentiation is particularly important in the case of rare, threatened, and highly localized seabirds^1,11^ as the life-history attributes of most seabirds render their populations robust in the face of fluctuations in breeding success, but highly sensitive to changes in adult mortality^12^. An estimated 95% of all seabird species breed in limited numbers of highly synchronous colonies^12,13^, rendering them susceptible to extreme breeding failure in the event of adverse environmental conditions or prey shortages. Nevertheless, poor reproductive output must be long-term and extensive to result in decreases in populations, whereas even slight changes in adult mortality can have lasting population or even species-level consequences^12^. Delayed sexual maturity and high philopatric tendencies also imply that seabird populations are typically slow to recover and that recovery after local extirpation is unlikely without interventions such as translocation or social attraction^12^. Thus, assuming local populations differ genetically, the loss of even a single population may result in the loss of important genetic variation that might ultimately affect the species’ ability to recolonize breeding sites, adapt to changing conditions, and possibly even to speciate^14,15^. Consequently, an understanding of the physical scales at which genetic differentiation occurs among populations is critical to the management of seabirds. This includes knowledge of their biology, ecology, and population trends including historical bottlenecks to identify population-specific management priorities.

Within the past few decades, seabirds have become exceptionally well-studied and knowledge of their overall conservations status and populations trends is considered more comprehensive than for any other group of marine organisms. However, Ref.^16^ assessed the population trends for the world’s seabirds and noted that as little as 19% of the global seabird population had been monitored more than five times between 1950 and 2010. Moreover, significant knowledge gaps exist with most colonies, and species in the tropics having been almost completely neglected. Among the most notable of the neglected species are the tropical petrels of the genera *Pseudobulweria* and *Pterodroma* (Procellariidae^11^). Most of these species breed on one or a few isolated islands and almost all remain little-known despite their poor conservation status. In this regard, Réunion Island (western Indian Ocean) is unique among tropical islands in that it supports two endemic species of petrel, the Mascarene (*Pseudobulweria aterrima*) and Barau’s (*Pterodroma baraui*) Petrels^17^. Both have unfavorable conservation status, suffering from threats imposed after the island was first colonized by humans in 1665^18^. These include light-induced mortality of fledglings and the predation of adults, eggs and chicks by invasive cats and rats^17^. The introduction and subsequent effects of these threats have increased dramatically following the rapid expansion of human activities during the last few decades. The Mascarene Petrel remains little-known, but over the last decade, considerable scientific attention has focused on the biology and conservation needs of the Barau’s Petrel. Despite this, a critical knowledge gap concerns contemporary and historic genetic diversity, population size, and population structure.

**Figure 1:**
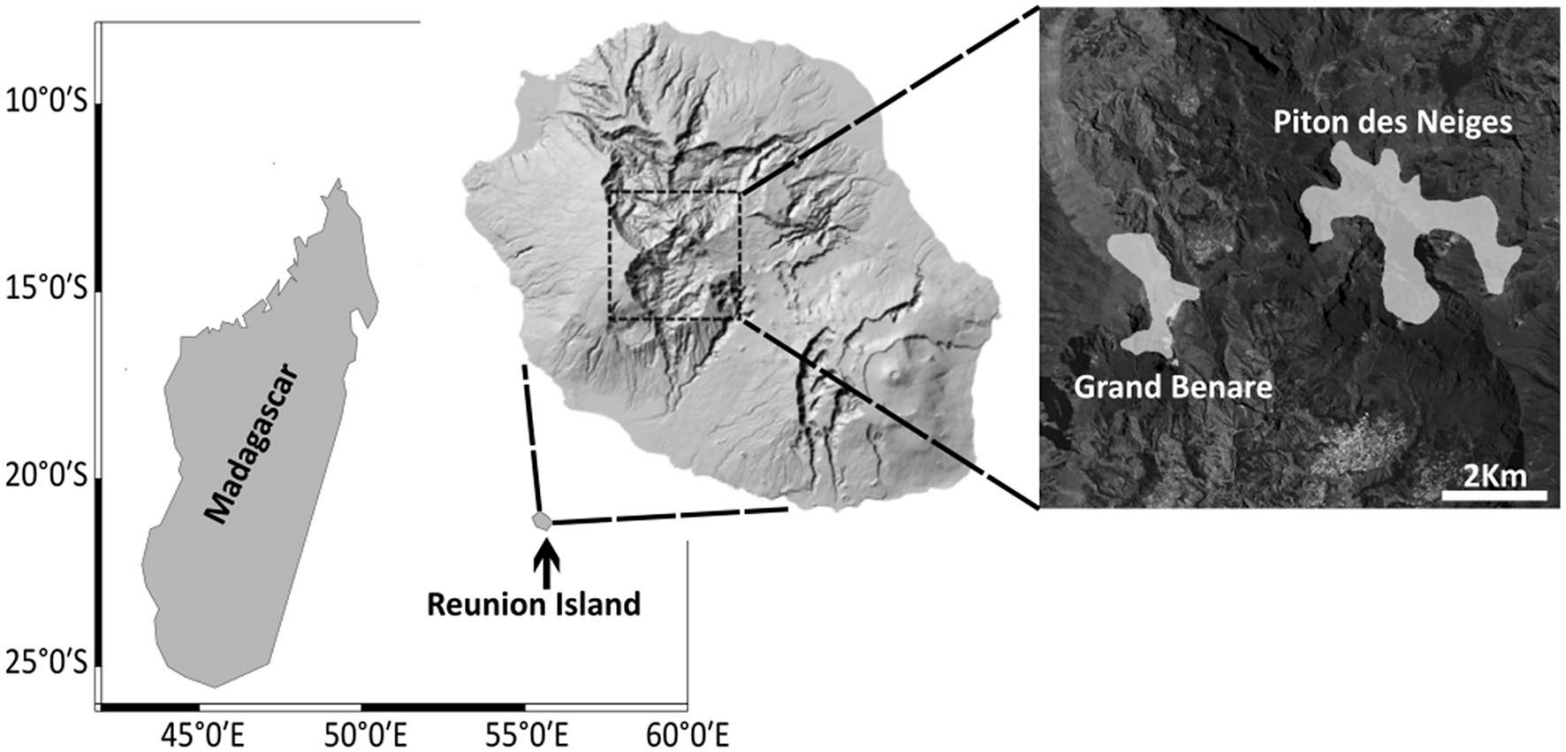
Approximate breeding distribution of Barau’s Petrel (*Pterodroma baraui*; shaded area) on the two central massifs of Réunion Island, Indian Ocean, highlighting the disjunct nature of the breeding colonies on the upper-most mountain slopes. Adapted from Ref.^17^. using satellite imagery from Google Earth Pro and spatially referenced using Ref.^79^. Location of the two Barau’s Petrel breeding colonies are shown in a three-part spatially referenced map.

To explore the links between intrinsic barriers to dispersal and genetic population structure, we employed diversity and structure analyses based on polymorphic microsatellites to investigate the genetic relationships between two proximate breeding colonies of the Barau’s Petrel separated by roughly 5 km on Réunion Island (Indian Ocean; Fig. 1). The overall objective was to explore the influence of the tendency for a high level of philopatry on population genetic divergence at a previously unexplored spatial scale and to understand the implications of this for the species’ conservation needs. We additionally tested for any recent changes to effective population size, phenotypic differences in morphology and, to quantify philopatry, we performed an analysis of band recoveries based on the long-term monitoring efforts on the Barau’s Petrel.

## Results

### Tests of assumption and indicators of genetic diversity

Successful microsatellite amplification was achieved across 14 loci in more than 94% of the sampled individuals. The fifteenth locus, PB_1030, was excluded due to a high number of false amplifications (see Primer Screening). Concerning indicators of genetic diversity across the 14 successfully amplified loci—the mean allelic diversity was roughly six alleles per locus. Both colonies contained private alleles amounting to 5% and 11% of total allelic diversity, carried by 7 and 25 individuals for the Grand Benare and Piton des Neiges colonies, respectively (Table 1). Means of N_A_ and A_E_ were significantly different in the global dataset (Wilcox. test: n = 14, w = 406, *p* value = 4.0 × 10^–6^) indicating a high proportion of rare alleles (allele frequency < 0.05) within each of the two colonies (39% and 47% of total allelic diversity for the Grand Benare and Piton des Neiges colonies, respectively). Most estimates of genetic diversity were higher for Piton des Neiges (Table 1), though the estimates of A_R_, based on a minimum sample size of 114 individuals, were not significantly different between the two colonies (*p* value = 0.59). Finally, the overall dataset showed no deviations from Hardy–Weinberg Equilibrium at the 99% confidence level (all *p* values > 0.01). Thus, the proportion of rare and private alleles in both populations was notable and suggestive of fine-scale genetic drift.

**Table 1:** Estimates of allelic diversity at two breeding colonies of the Barau’s Petrel (*Pterodroma baraui*), calculated across 14 polymorphic microsatellite loci.

Breeding colony	n	N_A_	A_R_*	PA	A_E_	H_O_	H_E_	F_IS_
Grand Benare	116.36 ± 0.34	5.79 ± 0.46^A^	5.77 ± 0.46^B^	4 (5%)	2.97 ± 0.29^C^	0.59 ± 0.04	0.62 ± 0.04	0.04 ± 0.03
Piton des Neiges	141.50 ± 0.23	6.21 ± 0.55^A^	6.09 ± 0.53^B^	10 (11%)	2.96 ± 0.31^C^	0.51 ± 0.06	0.59 ± 0.06	0.05 ± 0.02
Overall	128.93 ± 2.43	6.00 ± 0.35	6.15 ± 0.47	N/A	2.97 ± 0.21	0.57 ± 0.03	0.60 ± 0.04	0.05 ± 0.02

Indices of genetic diversity are as follows: n = mean number of individuals per locus ± S.E.; N_A_ = mean number of alleles per locus ± S.E.; A_R_ = mean allelic richness per locus ± S.E. (superscripts indicate statistical homogeneity among groups ^46^); PA = private allele richness (percentage of private alleles from total allelic richness in parenthesis); A_E_ = mean number of effective alleles per locus ± S.E.; H_O_ = mean observed heterozygosity over all loci ± s.e; H_E_ = mean unbiased expected heterozygosity ± S.E.; F_IS_ = mean fixation index ± S.E. calculated based on refs.^43,44^.

*Estimate based on a minimum sample size of 114 diploid individuals.

### Measures of genetic differentiation

The F_ST_ value (0.01; *p* value = 2.0 × 10^–3^) indicated weak though statistically significant genetic structure between the two breeding colonies. This was supported by two similar estimates of genetic differentiation: G′_ST_ (0.01; *p* value = 1.0 × 10^–3^) and R_ST_ (0.01; *p* value = 7.0 × 10^–3^), with 8 of the 14 loci showing *p* values < 0.05. The comparison between F_ST_ and R_ST_ was statistically non-significant (p*R*_*ST*_ = 8.0 × 10^–3^, *p* value = 0.28), indicating no major contribution of stepwise mutations to the observed genetic difference but rather genetic drift.

**Figure 2:**
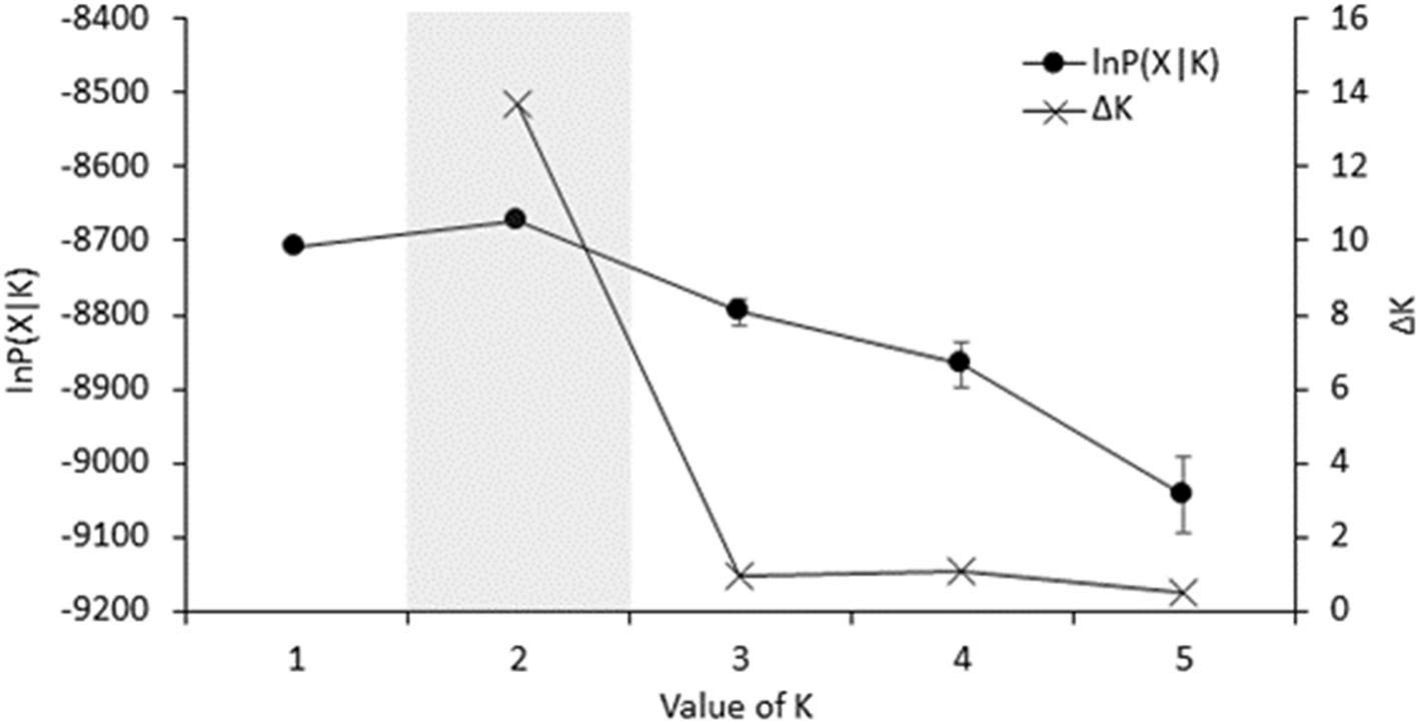
lnP(X|K; ± S.E.) and ΔK, as obtained in STRUCTURE version 2.3.3, with K ranging from 1 through 5. Burn-in period was set to 50,000 simulations followed by 100,000 repetitions. Each value was obtained by averaging the posterior probabilities of 10 independent runs. Shaded region highlights the optimal value of K based on both approaches. Estimation of the optimal number of genetic clusters based on the Bayesian clustering analysis performed in STRUCTURE and following two approaches^61,62^. The best-supported model was that of two genetic clusters as indicated by the maximum value of ΔK and the lowest standard error for lnP(X|K) at K = 2.

**Figure 3:**
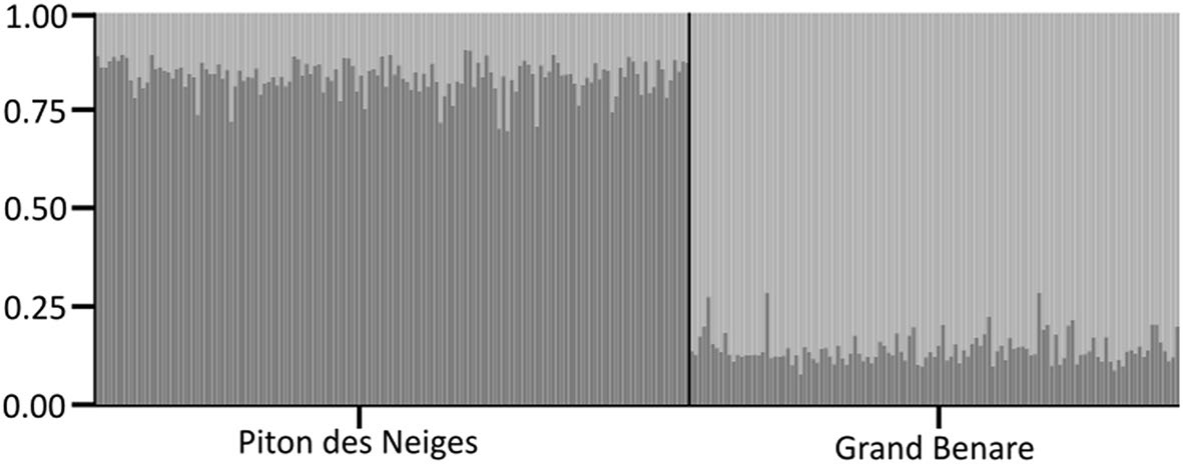
Membership coefficients assigned to 259 individual Barau’s Petrels, sampled at two breeding colonies, based on a Bayesian clustering analysis performed using an Admixture Model assuming K = 2 and using sampling location as a-priori information. Colors within each bar represent the two genetic clusters. Membership coefficients assigned to all individual Barau’s Petrels for the two genetic clusters. Vertical bars represent individuals and colors correspond to the two genetic clusters.

**Figure 4:**
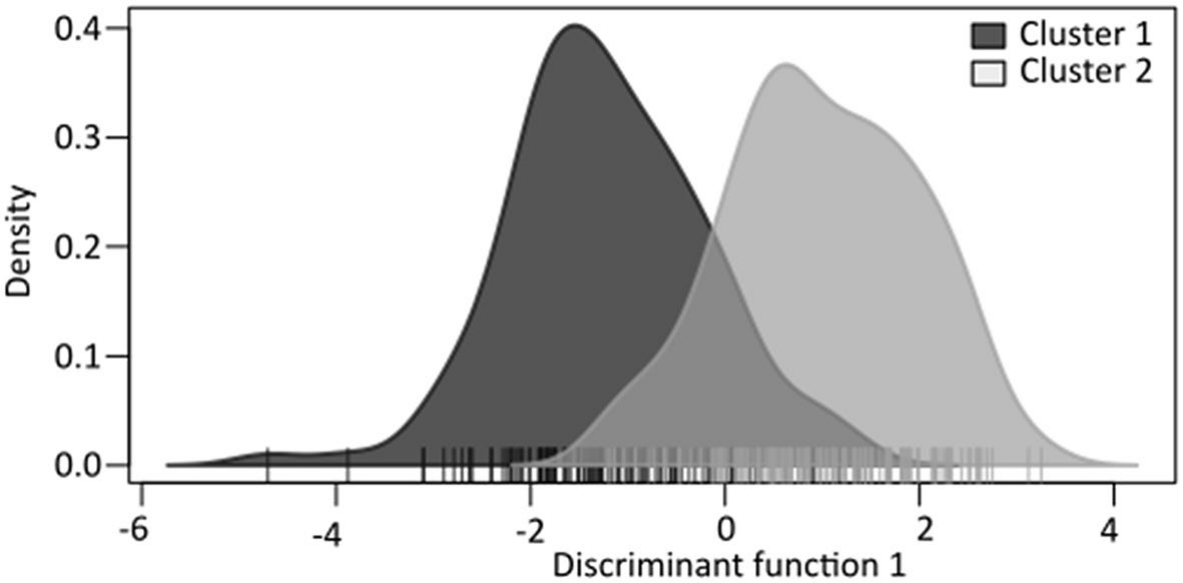
Density plot of Discriminant analysis of Principal Components (DAPC) highlighting clustering among 259 individual Barau’s Petrels, sampled at two breeding colonies, assuming K = 2 and using the first discriminant function and 80 principal components. Vertical bars represent individual assignments of 259 Barau’s Petrels from the Piton des Neiges (dark grey) and Grand Benare (pale grey) colonies. The densities of individuals are plotted along a given discriminant function, with different colors representing different groups. Here, the clear separation between Barau’s Petrels sampled at the Piton des Neiges and Grand Benare breeding colonies is visible.

Unexpectedly, following multiple approaches (Fig. 2), the best-supported model for the Bayesian clustering analysis was that of two genetic clusters using sampling location as a priori information (refer to “Methods” section). This was reinforced by the median value of L(K). The two genetic clusters correspond perfectly to the two breeding colonies, with individual membership coefficients averaging 0.85 ± 0.04 and 0.83 ± 0.04 for birds sampled from Grand Benare and Piton des Neiges, respectively (Fig. 3). The DAPC clustering procedure produced similar results to the STRUCTURE analysis, assuming K = 2 and using the first 80 principal components. Assignment probabilities of individuals sampled at Piton des Neiges averaged 0.79 ± 0.26 for Cluster 1, and assignment probabilities of individuals sampled at Grand Benare averaged 0.82 ± 0.23 for Cluster 2 (Fig. 4).

### Effective population size and test of bottleneck

The estimates of contemporary Ne are consistent with our evaluations of genetic diversity in the two colonies. Ne for the Piton des Neiges colony was estimated at 1148 [95% CI 434–infinite] and 1376 [95% CI 527–infinite] individuals at the cut-offs of 0.05 and 0.02, respectively. These were more than double the approximations for the Grand Benare colony where Ne was estimated as 514 (95% CI 269–2237) and 664 (95% CI 343–3541) individuals at the cut-offs of 0.05 and 0.02, respectively.

**Figure 5:**
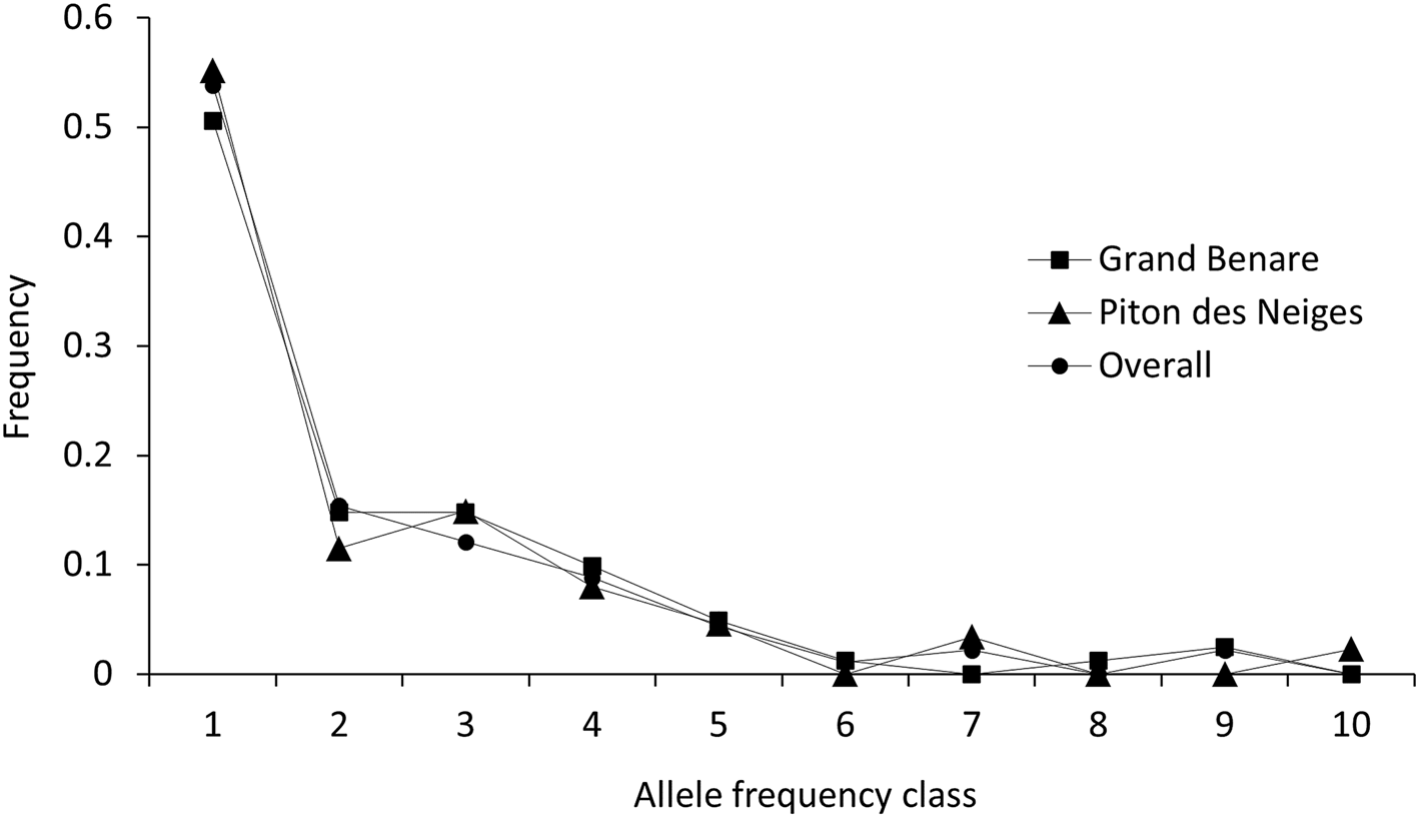
L-shaped mode shift highlighting allele frequency distributions and the absence of bottleneck in two breeding colonies and the overall sampled population of Barau’s Petrels (*Pterodroma baraui*). Population bottlenecks cause a characteristic mode-shift distortion in the distribution of allele frequencies. Bottlenecks cause alleles at low frequency (< 0.1) to become less abundant than alleles in one or more intermediate allele frequency class (e.g., 0.1–0.2). An L-shaped mode shift, as displayed above, is indicative of no recent bottlenecks for at least a few dozen generations.

The classic Bottleneck analysis based on the IAM, TPM and SMM models proved inconclusive in determining recent changes in Ne for the two breeding colonies or for the overall sampled population (Table 2). Nevertheless, the L-shaped allele distribution approach indicated that the two breeding colonies were in mutation-drift equilibrium suggesting that Ne has remained stable for at least a few dozen generations (Fig. 5). This is consistent with the M-ratio, which was high for Piton des Neiges (M = 0.94 ± 0.03) and Grand Benare (M = 0.96 ± 0.02) colonies as well as the overall dataset (M = 0.95 ± 0.03), suggesting that no severe population declines had occurred on recent timescales. Finally, using the coalescent likelihood MCMC approach, the estimates of the posterior distribution of past Ne revealed a relatively constant population size in the overall population for at least the last 5,000 generations (Current Theta index = 2.71, Intermediate Theta index = 2.68; Past Theta index = 2.49).

**Table 2:** P values from two tailed Wilcoxon sign-rank test of significance based on expected heterozygosity excess approach assuming mutation-drift equilibrium at the null hypothesis, and employing the Infinite Allele (IAM), Two-phase (TPM) and Stepwise Mutation (SMM) models.

	IAM	TPM	SMM
Piton des Neiges	0.03	0.09	0.01
Grand Benare	0.001	0.02	0.15
Overall	0.01	0.04	0.00

### Morphometric comparisons between the pair of colonies

**Figure 6:**
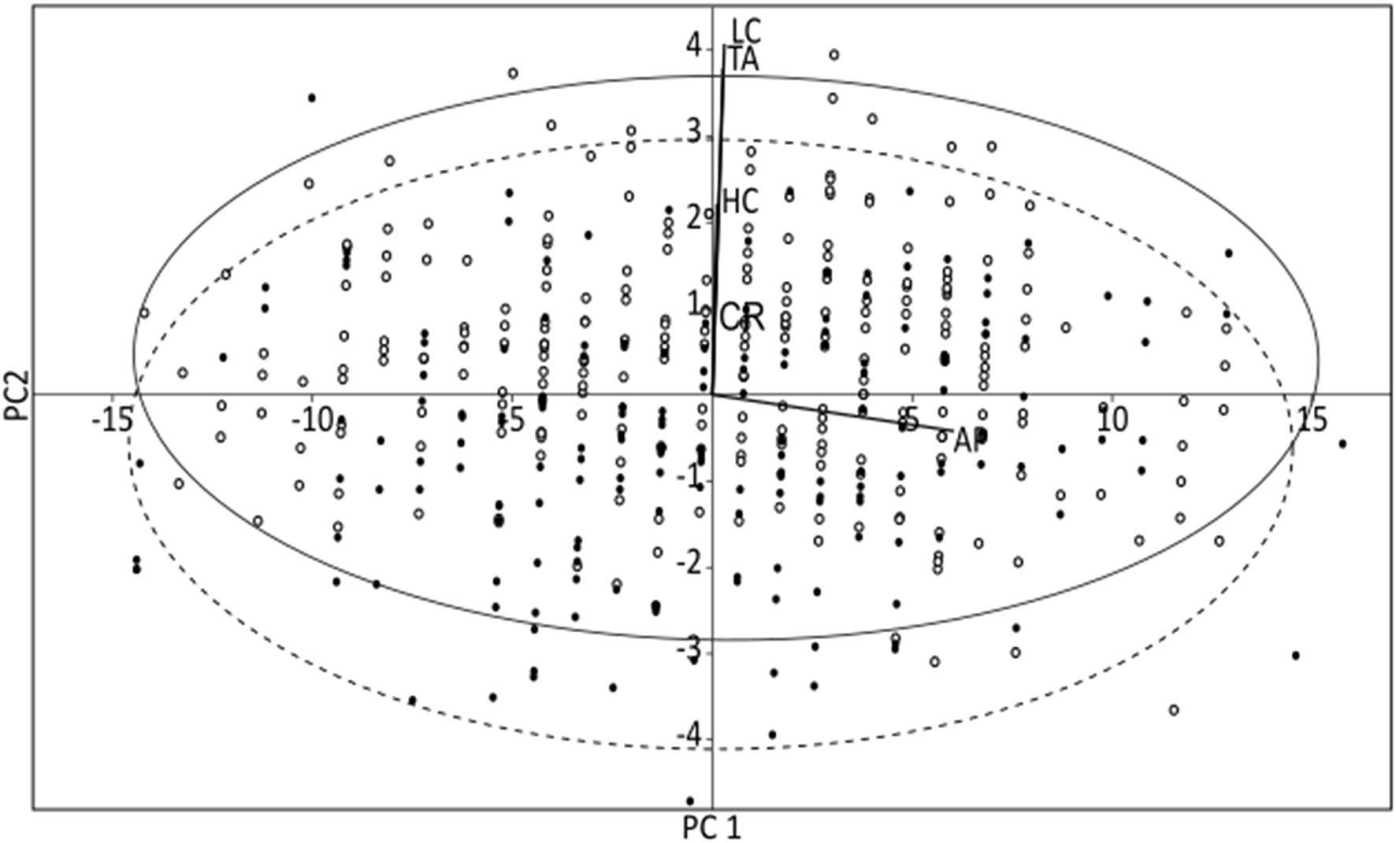
First and second axes of the Euclidian-distance standardized Principal Component Analysis, including standard 95% confidence ellipses, visualizing morphometric variation among adult Barau’s Petrels sampled at the Grand Benare (open circles and dashed ellipse; n = 180) and Piton des Neiges (filled circles and solid ellipse; n = 256) breeding colonies. Morphometric measurements are abbreviated in the biplot as follows: wing chord (AP), culmen length (LC), bill depth at the maximum gonydeal expansion (HC), maxillary unguis length (CR), and tarsus length (TA). Eigen values are as follows: PC1 = 35.56, PC2 = 2.11. Principal Component Analysis highlighting strong morphometric similarity among adult Barau’s Petrels, sampled at the Grand Benare and Piton des Neiges breeding colonies, using five standard measurements.

Morphometric measurements for adult Barau’s Petrels sampled in each of the two breeding colonies are presented in Table 3. One-way ANOSIM highlighted strong morphometric similarity among adult Barau’s Petrels sampled at the Grand Benare and Piton des Neiges breeding colonies (one-way ANOSIM: global R = 0.01, *p* value = 0.03). The significant *p* value linked to the very low R-value indicated that any difference between the pair of breeding colonies was the result of only a very small proportion of the measurements. The overlap among birds from the two breeding colonies is displayed in Fig. 6.

**Table 3:** Means of morphometric measurements (± S.E.) taken from adult Barau’s Petrels at the Grand Benare and Piton des Neiges breeding colonies, on Réunion Island (Indian Ocean).

	Grand Benare (n = 180)	Piton des Neiges (n = 256)	Overall (n = 436)
AP	293.00 ± 0.44	294.42 ± 0.37	294.23 ± 0.28
TA	38.02 ± 0.11	38.73 ± 0.07	38.44 ± 0.06
CR	19.14 ± 0.07	19.66 ± 0.04	19.45 ± 0.04
HC	12.22 ± 0.04	12.33 ± 0.03	12.28 ± 0.03
LC	32.73 ± 0.09	33.24 ± 0.07	33.03 ± 0.06

Morphometric measurements (in mm) are abbreviated as follows: wing chord (AP), culmen length (LC), bill depth at the maximum gonydeal expansion (HC), maxillary unguis length (CR), and tarsus length (TA).

### Banding recoveries within and between colonies

The long-term banding information is consistent with our estimates of genetic differentiation, indicating extremely high colony fidelity among 2842 individuals banded as both adults (1984) and pre-fledglings (858). More precisely, among 2690 re-sightings of banded birds, all were recovered from the same colony in which they were first banded. This is indicative of extremely high natal philopatry (no birds banded as fledglings in a given colony have been re-sighted as either prospecting or breeding adults at the other colony) and breeding site-fidelity (no birds banded as a breeding adults in a given colony have been re-sighted as a breeding or prospecting adult at the other colony).

## Discussion

The ‘seabird paradox,’ or the contradiction between the high vagility of seabirds and their apparent reluctance to disperse among breeding sites^19^, has drawn attention to the scale of population genetic differentiation, the mechanisms driving differentiation, and the implications these factors have for seabird conservation^4^. The scale of population genetic structure varies extensively among studied seabird species, down to extremely small spatial and temporal distances^20–22^, although the scale to which we observed population structure in the Barau’s Petrel is finer and more robust than has previously been recognized or explored (Table 4). The three indices of genetic differentiation highlighted that the Barau’s Petrel colonies constitute clear and distinct genetic populations each containing a proportion of unique genetic material, despite being separated by only 5 km. The comparison between F_ST_ and R_ST_ further highlighted that the influence of stepwise mutations was negligible compared to that of genetic drift when explaining differentiation between the two breeding colonies^23^. This unexpected result was supported by the banding information, DAPC and the Structure analyses, which together all indicated negligible admixture between the two breeding colonies. However, F_ST_ values greater than 0.05 are necessary to obtain reliable genetic estimates of migration among populations^24^. The two breeding colonies of the Barau’s Petrel can thus be considered as separate management units for conservation purposes and, comparing our estimates of Ne with the most recent population estimates (a total of 14,000 breeding pairs; Birdlife International), each genetic population contains a significant proportion of the species’ overall breeding population. Moreover, though sample size limited the accuracy of our estimates of Ne, the approximations for both breeding colonies are of a similar order of magnitude to the surveyed estimates of these populations: 800–850 pairs at Piton des Neiges, and 300–400 pairs at Grand Benare [Le Corre, unpub. Data].

**Table 4:** Estimates of population genetic structure based on microsatellite variation in nine diverse seabird species, by order of geographic distance between pairs of breeding colonies.

Species	Colonies	F_ST_	R_ST_	G_ST_	Number of microsatellites	Sample size per colony	Distance (km)	Refs.
Galapágos Petrel (*Pterodroma phaeopygia*)	Isabela/San Cristobal	0.14**	–	–	6	11/48	176	^9^
	Santiago/San Cristobal	0.12**	–	–	6	43/48	155	^9^
	Floreana/Santiago	0.17**	–	–	6	51/43	123	^9^
	Floreana/San Cristobal	0.09**	–	–	6	51/48	118	^9^
	Santa Cruz/San Cristobal	0.14**	–	–	6	53/48	98	^9^
	Isabela/Floreana	0.20**	–	–	6	11/51	86	^9^
	Isabela/Santa Cruz	0.23**	–	–	6	11/53	83	^9^
	Isabela/Santiago	0.07**	–	–	6	11/43	74	^9^
	Foreana/Santa Cruz	0.18**	–	–	6	51/53	72	^9^
	Santa Cruz/Santiago	0.26**	–	–	6	53/43	67	^9^
Hawaiian Petrel (*Pterodroma sandwichensis*)	Hawaii/Kaua'I	0.01*	–	0.02*	18	48/42	500	^25^
	Maui/Kaua'I	0.01*	–	0.01*	18	114/42	360	^25^
	Lanai/Kaua'I	0.27*	–	0.04*	18	28/42	300	^25^
	Hawaii/Lanai	0.02*	–	0.03*	18	48/28	195	^25^
	Hawaii/Maui	0.16*	–	0.02*	18	48/114	150	^25^
	Maui/Lanai	0.03*	–	0.05*	18	114/28	67	^25^
Shy Albatross (*Thalassarche cauta*)	Albatross/Pedra	0.19***	0.88**	–	6	20/20	400	^24^
	Albatross/Mewstone	0.05***	0.00	–	6	20/20	380	^24^
	Mewstone/Pedra	0.11***	0.08**	–	6	20/20	20	^24^
White-capped Albatross (*Thalassarche steadi*)	Logan Point/Disappointment	0.01	0.04	–	6	20/20	30	^24^
	SW Cape/Disappointment	0.00	0.00	–	6	19/20	25	^24^
	SW Cape/Logan Point	0.01*	0.02	–	6	19/20	6	^24^
White-tailed Tropicbird (*Phaethon lepturus*)	Mauritius/Réunion	0.02***	–	–	10	25/55	200	^76^
Brown Booby (*Sula leucogaster*)	Moleques/Cagarras	0.05	–	–	9	18/19	700	^77^
	Rocas/SPSP	0.62	–	–	9	19/24	671	^77^
	Abrolhos/Cagarras	0.06*	–	–	9	20/19	655	^77^
	FN/SPSP	0.60**	–	–	9	19/24	570	^77^
	Roacas/FN	0.07	–	–	9	19/19	153	^77^
Magnificent Frigatebird (*Fregata magnificens*)	Cabo Frio/Cagarras	0.00	–	–	8	14/9	125	^8^
European Shag (*Phalacrocorax aristotelis*)	BD/BB Scotland	0.01*	–	–	7	44/51	130	^78^
Barau’s Petrel (*Pterodroma baraui*)	Grand Benare/Piton des Neiges	0.01**	0.01**	0.01***	14	117/142	5	Current

Asterisks denote significant values as follows: *p < 0.05, **p < 0.01, ***p < 0.001.

In terms of the mechanisms of genetic population differentiation, our findings are clearly linked to the species’ extremely high philopatric tendencies, the apparent avoidance of cross-breeding between colonies and the absence of any other obvious barriers to genetic connectivity. A suite of individual, social, ecological, and historical factors are frequently invoked to explain patterns of genetic population differentiation in seabirds^3,4^. Characteristics such as biometrics and coloration are often of limited use given their similarity among different populations of seabirds^25^ and, in the case of the Barau’s Petrels, birds from the two breeding colonies are phenotypically indistinguishable. In addition—birds from our two colonies do not differ in either their breeding phenologies or coloration^25,26^, though each of these factors has been used to explain genetic population divergence in at least a few other species^21,27,28^. It has been hypothesized that non-visual reproductive signals including olfactory and acoustic cues may serve as better indicators of reproductive isolation in largely nocturnal seabirds^1^, although these are logistically and technically difficult to explore under field conditions and remain untested for most species^29^. Behaviorally, Barau’s Petrels are unique among small to medium sized petrels in that they arrive back at their colonies during daylight hours^17^. This is believed to be an energy-saving tactic, with birds using thermals to gain in altitude. However, recent radar surveys and thermal camera observations have shown that a large proportion of birds arrive at the colonies after sunset (authors’ unpublished data), and the peak activity around the colonies happens after dark as in other related species. Thus, daytime arrival at colonies is unlikely to play a role in cross-colony interactions and colony partitioning. Moreover, several molecular studies have suggested that contemporary gene flow may not immediately override the influence of historical population genetic structure^3^. In the case of the Barau’s Petrel, however, there is no evidence of historical range contractions or recent bottlenecks and the major threats the species faces (e.g. light-induced mortality, predation by invasive mammals^17^;) are all relatively recent and linked to man’s colonization and expansion on Réunion Island^18^. This implies that the main threats to Barau’s Petrel populations may not yet have affected genetic diversity in this long-lived seabird. Nevertheless, the influence of small population size on genetic diversity cannot be neglected and, although our results highlight good genetic diversity with no evidence of inbreeding, both colonies contain rare and private alleles which are likely to disappear if these threats are maintained.

Some seabirds exhibit population genetic structure in the absence of recognizable barriers to gene flow, suggesting that selective or social processes including philopatry can limit gene flow. Indeed, philopatry has been recognized as the second most obvious potential barrier to gene flow in seabirds after the physical isolation of breeding populations^3^. Given sufficient time, philopatry has the potential to restrict gene flow sufficiently to lead to reproductive isolation^5^, and has contributed to total speciation among populations of seabirds in a handful of examples^9,30,31^. However, philopatry alone may be insufficient to result in complete reproductive isolation as it usually acts in combination with other barriers to gene flow. In the case of the Barau’s Petrel, the genetic implications of the species’ philopatric tendencies appear to be exacerbated by high mate fidelity^32^. Barau’s Petrels nest in high altitude elfin forest above 2400 m a.s.l. on the two isolated peaks of Réunion Island^23^. The locations of the colonies are determined primarily by habitat conditions, particularly where soil conditions are suitable for burrow construction. The colony on Piton des Neiges is the largest known colony, situated in an area of optimal habitat conditions around 2400 m a.s.l., whereas the habitat on Grand Benare ranges from optimal to sub-optimal between the altitudes of 2600 m a.s.l. and 2800 m a.s.l. Thus, local environmental factors may additionally inhibit effective dispersal between the two breeding colonies, through the isolation of the nesting habitats and the habitat selectivity of the birds themselves. Importantly, however, it is not yet known whether Barau’s Petrels will engage in subadult prospecting at non-natal colonies though the limited number of band recoveries of fledglings at colonies has yet to detect such behavior and colony exchanges of breeding adults appears to be genetically negligible. Thus, although philopatry may not be universal among all seabird lineages^33^, the extremely fine-scale at which it obviously contributes to genetic population divergence in the Barau’s Petrel further emphasizes its role as an intrinsic barrier in the evolution of seabird diversity and endemism.

Our results highlight that genetic studies below the taxonomic level of species are critical to the practice of applied conservation. Seabirds are disproportionately represented among the birds that are most at risk of extinction^11^. Their highly adapted biology reduces the capacity of populations to absorb additive mortality, particularly of adults, and slows their potential for recovery^1,11^. Moreover, the breeding distribution of many species is restricted to a limited number of sites, often on single islands or archipelagos, where populations can rapidly diverge^11,25^. The loss of genetic diversity within sub-divided populations can therefore have lasting impacts on the ability of the species to adapt, and possibly also to speciate. In practice, this means that seabird populations should be maintained across all conservation units to retain genetic variation at the species level and to protect the potential for future speciation. In addition to the genetic implications, the philopatric tendencies of most seabirds further imply that, without intervention (e.g. translocation or social attraction), a species would be slow to recover or repopulate areas following local extirpation^34^. This reinforces the fact that modern molecular approaches and population-level studies provide an invaluable perspective from which to view and delimit the appropriate units for management, offering powerful approaches for the assessment of the effects of disturbance on populations to the guidance of long-term recovery programs.

## Materials and methods

### Sample collection and microsatellite amplification

This study was carried out in compliance with the ARRIVE guidelines. Fieldwork was performed between the austral summers of 2008/2009 and 2012/2013 at two large breeding colonies of the Barau’s Petrel on Réunion Island, Indian Ocean (Fig. 1). Breeding is highly synchronous between the two colonies, with no differences in foraging behavior during either the breeding or non-breeding periods^26,35^. A total of 259 adult birds were captured at their nests in the Grand Benare (n = 117) and Piton des Neiges (n = 142) colonies. A blood sample of approximately 0.2 ml was collected from each bird through medial metatarsal venipuncture and stored in 70% ethanol for further processing. All procedures were performed in accordance with relevant guidelines and regulations. We also received ethical approval from the Animal Ethics Committee (ZOOL-01-2013) at Rhodes University (South Africa), the Université de La Réunion (Réunion Island), Parc National de La Réunion, the Ethical Committee of Réunion Island, and the Centre de Recherche sur la Biologie des Populations d’Oiseaux (CRBPO, personal program of Matthieu Le Corre, PP6109). DNA was subsequently extracted from whole blood subsamples using the DNeasy Blood and Tissue extraction Kit (Qiagen).

A classic 3-primer Polymerase Chain Reaction (PCR) approach was used to amplify DNA at fifteen microsatellite loci following ref.^36^, alongside four fluorescently labelled dyes (6-FAM, PET, VIC, and NED) for the universal M13 forward primer enabling fragment analysis multiplexing^37^. PCR product sizes were determined using a 3730XL DNA analyzer (Applied Biosystems) by Gentyane platform (Clermont-Ferrand, France) and were estimated with the LIZ(500) standard using GeneMapper version 4.0 (Applied Biosystems).

### Primer screening

Successful microsatellite amplification was achieved across the 15 loci in more than 55% of all samples from the two breeding colonies of the Barau’s Petrel. Around 3.09% of the global dataset consisted of null values. Locus PB_1030 contained an extremely high percentage of missing data with 31.62% and 47.18% missing data for Grand Benare and Piton des Neiges, respectively. Removal of this locus for all further analyses reduced the proportion of missing data to within acceptable limits (a maximum of 2.56% at any one locus in either colony). The resulting genotype data were checked for amplification errors and the presence of null alleles using MicroChecker 2.2.3^38^. The only examples of stuttering were at loci PB_2742 and PB_3916, indicated by the highly significant shortage of heterozygote genotypes with alleles of one repeat unit difference. Null allele frequency was low and within acceptable limits, though null alleles were present for loci PB_1890, PB_2742, and PB_4708 in both colonies, and PB_3916 only in the Grand Benare colony. All loci for which null alleles were detected were associated with homozygote excesses. Each sample-locus combination was tested for linkage disequilibrium using GenePop 4.0.10^39^, employing the exact probability test (Markov chain parameters: 10,000 dememorizations, 100 batches, 1000 iterations per batch), and with False Discovery Rate correction^40^. Presence/absence of linkage disequilibrium was confirmed with the method of Index of Association^41^, in the R package poppr^42^. No linkage disequilibrium was observed among any of the loci (all *p* values not significant) using either approach.

### Estimates of genetic diversity

Estimators of genetic variability were calculated across the 14 successfully amplified loci including the allele frequencies at every locus, the average number of alleles per locus (N_A_), the average number of effective alleles (A_E_), private allele richness (PA), and the observed (H_O_) and unbiased expected (H_E_) heterozygosities according to Ref.^43^ using GenALEx 6.5^44^. The statistical significance of differences between N_A_ and A_E_ were assessed using non-parametric Wilcoxon Signed Rank tests implemented in PAST 3.1.5^45^. Allelic richness (A_R_;^46^), adjusted for discrepancies in sample size using rarefaction, was additionally calculated for each breeding colony using FSTAT 2.9.3^47^, and compared between the colonies using 10,000 permutations. Deviations from the Hardy–Weinberg Equilibrium (HWE) were assessed with the R package pegas^48^, using the exact test based on 1000 Monte Carlo permutations and a χ^2^ test for global HWE. Finally, we estimated Wright’s inbreeding coefficient (F_IS_^49,50^) according to Ref.^51^ using GenAlEx version 6.5^44^.

### Genetic differentiation and structure

Wright’s overall multi-locus fixation index (*F*_*ST*_^49^), and its associated *p* value, were computed over all 14 loci to assess genetic differentiation following ref.^45^, using the R package adegenet^52^. The statistical significance of differences between the pair of breeding colonies in this index, was tested using 10,000 random permutations. Mutation processes occur at relatively high rates and with stepwise changes in allele sizes at microsatellite loci, which introduces bias into classical measures of population differentiation such as F_ST_^53^. Thus, to corroborate these results, we calculated two additional measures of genetic differentiation: *R*_*ST*_ using SPAGeDi^53^, and *G*′_*ST*_ using GenoDive 3.04^54^. R_ST_ uses a stepwise mutation model to assess variances in allele sizes rather than allele frequencies (as in F_ST_) and better reflects population differentiation among microsatellite loci in instances when stuttering is observed^55^. G′_ST_ is equivalent to F_ST_ but with different statistical properties^56^, and with correction for bias stemming from sampling a limited number of populations^57^. The simple test of permutated *R*_*ST*_ (*pR*_*ST*_), using 10,000 permutations of the genotypes, was additionally used to determine whether F_ST_ equalled R_ST_ and to assess whether stepwise-like mutations or genetic drift contributed to genetic differentiation^53^.

The multilocus genotype data were additionally used to perform a Bayesian clustering analysis implemented in STRUCTURE 2.3.4^58^. Multiple independent simulations, using a burn-in period of 50,000 simulations followed by 100,000 repetitions, were first run using different settings (e.g. admixture versus no-admixture model, LOCPRIOR verses no-LOCPRIOR) to assess convergence. The Dirichlet parameter (ɑ) was used to determine whether the standard admixture model was appropriate following Refs.^58,59^. Similarly, the r-index was used to determine the informativeness of the sampling location (LOCPRIOR), with low values of *r* indicating that sampling locations are informative to the overall model^60^. An admixture model (ɑ = 3.26 ± 0.57^58, 59^), assuming sampling location (LOCPRIOR; r = 0.74 ± 0.12^60^), was most appropriate and provided the best levels of convergence. Correlated allele frequencies were assumed^58,60^. The number of genetic clusters (termed K) was subsequently determined following three criteria: (1) the log likelihood given K (lnP[X|K]^61^), (2) the second-order rate of change of mean log-likelihood (ΔK^62^) and (3) the median value of L(K). The first two were calculated using STRUCTURE HARVESTER online Web server^63^, while the third was calculated using CLUMPAK^64^. Ten independent simulations, at the optimal value of K, were then run using 1,000,000 iterations each (after a burn-in of 500,000 steps). Finally, CLUMPAK^64^ was used to find the optimal individual alignments of replicated cluster analyses and to plot the estimates of individual membership for all genetic clusters.

Lastly, population structure was explored using Discriminant Analysis of Principal Components (DAPC^65^). This approach does not make any assumptions about HWE or linkage disequilibrium but is sensitive to fine genetic differences among populations. We used K-means clustering of principal components for K = 1 to K = 5 and Bayesian Information Criteria (BICs) to assess the optimal number of genetic clusters. The value of K with the lowest BIC value was considered optimal^66^. DAPC was applied using the Adegenet package 2.1.1 in R (52).

### Effective population size and test of bottleneck

Contemporary estimates of effective population size (*Ne*) based on the 14 microsatellite markers were calculated for the two Barau’s Petrel breeding colonies using the molecular co-ancestry method of Ref.^67^, as implemented in NeEstimator 2.0^68^. *Ne* was calculated assuming a monogamous mating pattern^32^. Two allele frequencies were adopted as conservative cut-offs to minimize the effect of the presence of rare alleles^68^: 0.02 and 0.05. Estimates of 95% confidence interval (CI) were calculated by jackknifing over loci for each estimate.

Finally, we also used Bottleneck 1.2.02^69^ to test for recent changes in population size using the heterozygosity excess approach, assuming mutation-drift equilibrium as the null hypothesis. We used the Infinite Allele (IAM), two-phase (TPM; non-stepwise = 0.22%, variance = 12; typical values for many microsatellite markers^70^, and stepwise mutation models (SMM) based on 10,000 replications and the Wilcoxon sign-rank test of significance (two tailed for heterozygosity excess or deficiency). These results were confirmed with the L-shaped method of allele mutation-drift equilibrium^71^, the M-ratio calculated (mean ± S.E.) across all 14 loci using the R package StrataG 2.4.905^72,73^, and the approximate likelihood MCMC approach using modelled Theta values (Θ = 4Ne × Mu; where Ne is the effective population size and Mu is the microsatellite mutation ratio) using the VarEff 1.2 R package^74^.

### Banding information and morphometric measurements

Annual monitoring at the Grand Benare and Piton des Neiges breeding colonies began over the austral summers of 2007/2008 and 2002/2003, respectively. Subsequently, the two colonies have been visited at least twice annually up to the present for banding and control of breeding adults (November/December) and for banding of pre-fledging chicks (March). All re-sightings to the present day (13 and 18 years of data for the Grand Benare and Piton des Neiges colonies, respectively), were collated to quantify philopatry and levels of individual exchange between colonies. Re-sightings of birds banded at two age groups (adults, pre-fledglings), within and between each breeding colony, were determined using dplyr 1.0.2^75^ in R.

Morphometric measurements of adult Barau’s Petrels were taken from a sample of individuals and compared between the two breeding colonies using One-way Analysis of Similarities (ANOSIM) based on an Euclidean distance measure and 9999 permutations in PAST 3.1.5^45^. Barau’s Petrels are morphologically indistinguishable between the sexes^32^, and thus all individuals were pooled for the purposes of this analysis so as not to limit our ability to detect differences between the two colonies. Morphometric measurements were taken in the field with 1 mm precision and included the following: wing chord (AP), culmen length (LC), bill depth at the maximum gonydeal expansion (HC), maxillary unguis length (CR), and tarsus lengths (TA).

## Supplementary Information

Supplementary information.

## Data Availability

All data needed to evaluate the conclusions in the paper are present in the paper and/or the Supplementary Information. Raw microsatellite genotypes of the 259 individual Barau’s Petrels, for all 15 loci, are available in the supporting information (Table S1).
